# Interobserver Agreement and Plane-Dependent Intraobserver Variability of Shear Wave Sonoelastography in the Differential Diagnosis of Ectopic Thymus Tissue

**DOI:** 10.3390/jcm10020214

**Published:** 2021-01-09

**Authors:** Zbigniew Adamczewski, Magdalena Stasiak, Bartłomiej Stasiak, Magdalena Adamczewska, Andrzej Lewiński

**Affiliations:** 1 Department of Endocrinology and Metabolic Diseases, Polish Mother’s Memorial Hospital Research Institute, 93-338 Lodz, Poland; zbigniew.adamczewski@umed.lodz.pl (Z.A.); mstasiak33@gmail.com (M.S.); 2 Department of Endocrinology and Metabolic Diseases, Medical University of Lodz, 93-338 Lodz, Poland; magdalena.adamczewska1@stud.umed.lodz.pl; 3 Institute of Information Technology, Lodz University of Technology, 90-924 Lodz, Poland; bartlomiej.stasiak@p.lodz.pl

**Keywords:** shear wave elastography, ectopic thymus, thyroid, ultrasound, interobserver variability

## Abstract

Shear wave elastography (SWE) has been demonstrated to be a useful tool in the differential diagnosis of ectopic thymus tissues (ETs), providing quantitative values of the shear wave stiffness (SWS) of both ETs and adjacent thyroid tissue. However, no data are available on the potential influence of the imaging plane (transverse vs. longitudinal) on the obtained SWS and shear wave ratio (SWR) values in SWE of these tissues. Moreover, no reports on the interobserver repeatability of SWE were published in regard to ETs. The aim of this study has been to evaluate the potential influence of the examination plane—transverse vs. longitudinal—on the SWS and SWR results, as well as to determine whether SWE of ETs is subjected to interobserver variability. SWE was demonstrated to have high inter- and intraobserver agreement in the evaluation of ETs and adjacent thyroid tissue. Significant differences between SWS values, but not SWR values, obtained in the transverse and longitudinal planes were observed. This phenomenon is probably a result of anisotropy-related artifacts and does not reduce the reliability of the method. SWE operators should be aware of the presence of plane-dependent artifacts to properly interpret the obtained results.

## 1. Introduction

Ultrasonography (US) is a readily available, noninvasive tool used in neck imaging and the first step in the diagnostic algorithm of thyroid nodules [1,2]. However, US results highly depend on the interpretation and experience of the operator, with the sensitivity and specificity values ranging between 65.3 and 81.9%, and 60.7 and 68.9%, respectively [3]. Thus, some new tools based on US have been introduced for the improvement of diagnostic capabilities. Sonoelastography provides valuable information about lesion stiffness/elasticity. Malignant thyroid lesions are known to be much stiffer than benign lesions [4,5]. The first commonly available method was strain elastography (SE), which is based on a stiffness comparison of the analyzed lesion and the adjacent healthy tissue, providing the result in the form of a strain ratio only. This tool provides semiquantitative analysis but does not allow precisely assessing the elasticity of the particular lesion. Moreover, it appeared to be researcher-dependent, as the result is associated with the degree of tissue compression [6,7]. Therefore, the use of strain elastography (SE) in US diagnosis raises much controversy. Except for supporters of this method [8], there are many opponents who do not accept the difficulties in obtaining an artifact-free result [9,10]. To avoid the limitations of SE, shear wave elastography (SWE) has been introduced. This examination is conducted without manual compression, and the result is expressed as a quantitative elasticity value (kPa). These promising properties of SWE allow us to believe that this method is accurate, operator-independent and reproducible. Such an assumption requires confirmation in the studies performed on specific tissues and organs.

Most of the studies focused on SWE concern the discrimination between benign and malignant lesions or the diagnosis of diffuse organ diseases, e.g., liver fibrosis or thyroiditis [11,12]. Only limited research has been performed to determine in vivo values for healthy soft tissues [13]. None of the published studies have included ectopic thymus tissues (ETs).

Recently, young children and even infants have been frequently referred for neck US because of enlarged lymph nodes or other palpable lumps in the neck. Ultrasound is also performed as part of a population study and due to a family history of thyroid disorders. The increasing availability of the ultrasound procedure leads to a new phenomenon of the incidental detection of various types of intrathyroidal and extrathyroidal lesions in children [14].

Any thyroid lesion found in a child should raise alertness and require further evaluation, as the frequency of thyroid cancer in thyroid lesions in children is much higher than in adults [14]. The most common benign neck lesions that can mimic malignancy are ETs [15]. The incidence of ectopic thymus in the neck in children was reported from 0.99% [16] to 1.8% [17]. Intrathyroidal ETs (IETs) and extrathyroidal ETs (EETs) resemble thyroid carcinoma and metastatic lymph nodes, respectively [15]. Proper differential diagnosis of such lesions allows avoiding unnecessary invasive procedures, including surgery. Quantitative tissue assessments in normal parenchymal organs (such as thyroid or thymus) using SWE are rarely conducted [13]. Therefore, it is remarkably important for the examiner to be familiar with normal shear wave stiffness (SWS) ranges to be able to differentiate pathological (neoplastic or inflammatory) and benign lesions.

Our research group demonstrated the usefulness of SE in the differential diagnosis of ETs [15]. However, due to the significant limitations of the method, we recently analyzed the application of SWE in children with ETs. SWE has appeared to be a useful tool in this indication, providing quantitative values of the SWS of both ETs and adjacent thyroid tissue and computing shear wave ratio (SWR), defined as a quotient of these values [18]. However, no data are available on the potential influence of the imaging plane (transverse vs. longitudinal) on the obtained SWS and SWR values in SWE of the neck tissues. Moreover, there are few studies evaluating the interobserver repeatability of SWE, and none of them include ETs or have been performed in a pediatric population [19,20].

The aim of this study was to evaluate the potential influence of the examination technique—transverse vs. longitudinal plane—on the SWS and SWR results obtained in healthy children with ETs. The aim of this research has also been to determine whether this SWE is subjected to variability between two independent researchers (interobserver variability).

## 2. Experimental Section

### 2.1. Patient Selection and Diagnostic Procedures

Fifty consecutive cytologically confirmed ETs found in 28 children who were referred to our department because of suspicion of papillary thyroid carcinoma (PTC)/neoplastic lymph nodes were included in the study. Among the 50 lesions, 33 were IETs and 17 were EETs. To exclude any thyroid disease, in all patients, laboratory tests were performed, including thyrotropin (TSH), free thyroxine (FT4), free triiodothyronine (FT3), antithyroid peroxidase antibodies (aTPO), antithyroglobulin antibodies (aTg) and TSH receptor antibodies (TRAb). All parameters were measured by electrochemiluminescence immunoassay (ECLIA) with the Cobas e601 analyzer (Roche Diagnostics, Florham Park, NJ, USA). On the basis of the laboratory results, 3 children with positive aTPO and hypothyroidism, as well as one child with Graves’ disease, were excluded from the initial number of 32 children. Only children without any abnormal thyroid function and/or autoimmune disorders were included in the study. In all patients, US-guided fine-needle aspiration biopsy (FNAB) was performed under moderate sedation or general anesthesia. Cytological smears were evaluated by the same high-volume pathologist. The smears including only small lymphocytes with scattered epithelioid cells (without the presence of macrophages, plasma cells, eosinophils, histiocytes or other cell types) were diagnosed as ETs. Only lesions with cytological confirmation of ET were included in the study.

After more than 3 months from the FNAB procedure, US and SWE neck examinations were performed with a 4–15 MHz linear transducer (Aixplorer MACH30, SuperSonic Imagine, Aix en Provence, France). The neck was in a natural position to avoid hyperextension. During the SWE examination, the probe was applied gently to the skin with a gel thick coating so as to avoid direct contact between the probe and the neck. The US and SWE examinations were carried out by two independent examiners—endocrinologists with more than 10 years of experience in US and elastography of the thyroid gland (M.S.—Researcher 1; Z.A.—Researcher 2).

During the SWE examination, a single researcher performed 4 measurements in the IET group (thyroid and IET in the transverse and longitudinal planes) and 3 measurements in the EET group (EET in the transverse and longitudinal planes, thyroid in the longitudinal plane). All SWS measurements were recorded in kilopascals. SWR values were calculated for thyroid/IET pairs in both planes—longitudinal and transverse—and for thyroid/EET pairs in the longitudinal plane only, as there was no possibility to present the EET and some corresponding thyroid tissue in a single transverse plane. All children had the SWE examination carried out two times (by the two researchers) in a single session. The researchers were blinded to each other’s results. No statistically significant association between patient’s age and SWS of either IET or EET was previously observed [18]; thus, we did not include age into the statistical analysis.

### 2.2. Statistical Analysis

The analyzed variables were quantitative; they were described by the mean (as a measure of position) and standard deviation (as a measure of dispersion). Inter- and intraobserver variability of the analyzed measurements were characterized as both the absolute difference (D) and the relative difference expressed as a percentage of the absolute difference with respect to the mean value of the compared measurements (D%). Their distributions were described using the mean value together with the 95% confidence interval (95% CI). The distributions of most variables were not normal, hence the hypotheses about the equality of distributions were tested using a nonparametric method, the Wilcoxon signed-rank test. For the verification of statistical hypotheses, a value of *p* < 0.05 was considered statistically significant. To further evaluate interobserver agreement, intraclass correlation coefficients (ICC) (2, 1) were calculated, with the following interpretations: <0.40—poor; 0.40–0.59—fair; 0.60–0.74—good; 0.75–1.00—excellent [21].

All the analyses were performed using the Statistica 13.3 software (TIBCO Software Inc., Palo Alto, CA, USA).

## 3. Results

### 3.1. Interobserver Variability

**Figure 1:**
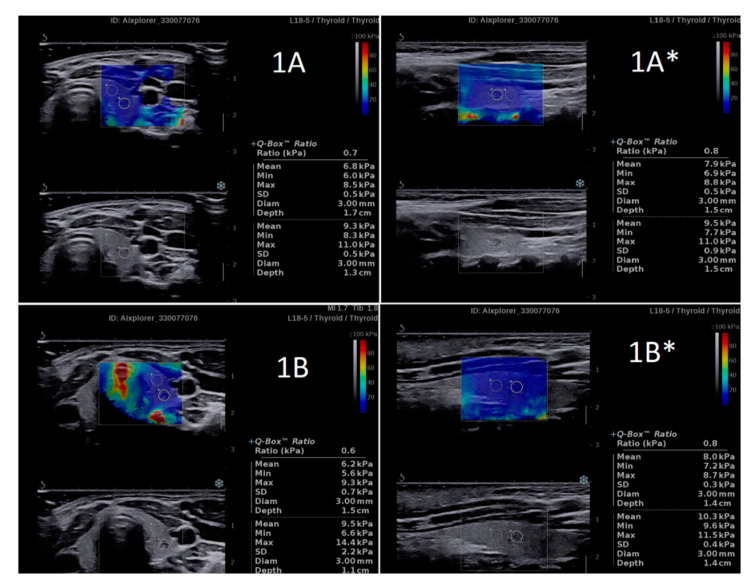
An example of the SWS and SWR results obtained by two independent researchers ((**A**) R1 and (**B**) R2—Patient 1. A high similarity is clearly visible for both the SWS and SWR values. * Longitudinal plane imaging. SWR, shear wave ratio; SWS, shear wave stiffness.

**Figure 2:**
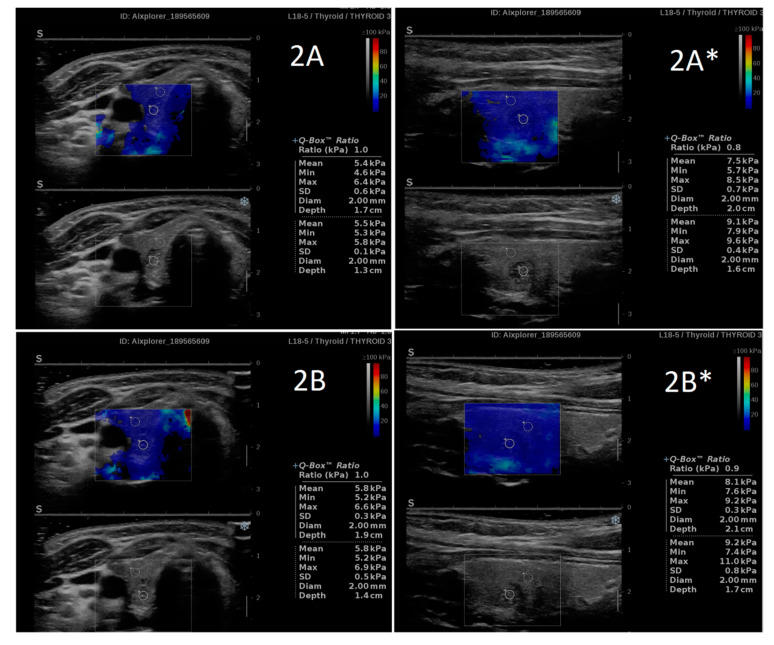
An example of the SWS and SWR results obtained by two independent researchers ((**A**,**B**) Patient 2). A high similarity is clearly visible for both the SWS and SWR values. * Longitudinal plane imaging.

The comparison between SWS and SWR results obtained by two independent researchers revealed high similarity. No statistically significant differences were found between SWS values in ETs (in IETs, EETs and both considered jointly) as well as in adjacent thyroid tissue, separately analyzed in the transverse plane and in the longitudinal plane (Table 1, Figure 1 and Figure 2). Similarly, no differences between the researchers were found in SWR values analyzed in both planes (Table 1, Figure 1 and Figure 2).

The ICC values computed for SWS of ETs and SWS of the thyroid in the transverse plane were 0.76 and 0.81, respectively, and 0.86 and 0.84 for the longitudinal plane, respectively.

### 3.2. Intraobserver Variability Related to the Imaging Plane

**Figure 3:**
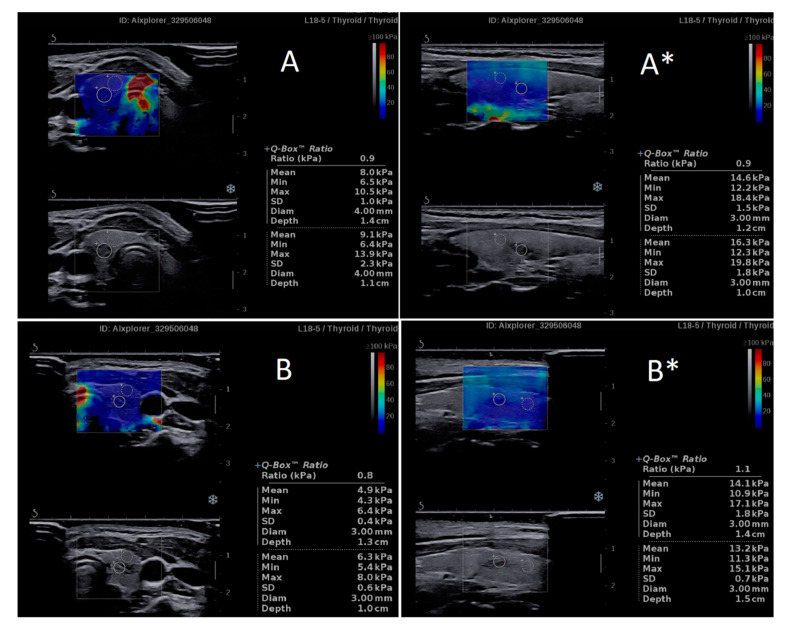
Examples of the significant difference in shear wave stiffness (SWS) values of two ectopic thymuses (**A**,**B**) and adjacent thyroid tissue between the transverse and longitudinal plane imaging, simultaneously with similar shear wave ratio (SWR) values, regardless of the plane. * Longitudinal plane imaging.

Highly significant differences were found in SWS values between transverse and longitudinal plane imaging. The values obtained in the longitudinal plane were higher than those observed in the transverse plane (Figure 3, Table 2 and Table 3). Significant differences were found for all evaluated structures, including IETs, EETs (Table 2) and thyroid tissue adjacent to ETs (Table 3). However, no differences were found in the SWR values obtained in both imaging planes (Figure 2 and Figure 3, Table 4).

## 4. Discussion

The finding of a pathological lesion in a child’s neck should arouse diagnostic alertness. Frequently, US examination is not sufficient to provide an unequivocal decision on whether the lesion is really suspicious. The differential diagnosis of ETs is an accurate example of such a situation, as benign ETs often resemble malignant lesions in US. Ectopic thymic tissues can be found in children only, and FNAB is possible to be performed in this group, mainly in sedation or general anesthesia. Thus, there is a need for an accurate, quick and simple diagnostic method for differentiation between actually suspicious lesions requiring immediate FNAB and IETs/EETs, which can be followed up for some time. In the latter case, FNAB can even be avoided in some children, especially those with some relative contraindications for anesthesia [15]. The utility of SWE in the differential diagnosis of ETs has recently been proven [18]. However, the potential influence of the imaging plane (transverse vs. longitudinal) and the interobserver variability on the obtained SWS and SWR values in SWE of the ETs and adjacent thyroid tissues has never been analyzed. Both factors can potentially modulate SWE results. Thus, the evaluation of their significance seems mandatory for the unequivocal recommendation of SWE as a reliable and researcher-independent method.

In the present study, a high interobserver agreement was demonstrated. We did not find significant differences between SWS measured by both the researchers regardless of the plane. Moreover, no differences between the examiners were found in the SWR values analyzed in both planes. A significant similarity of the SWS results was obtained in ETs (in IETs, EETs and both considered jointly) as well as in adjacent thyroid tissue. The highest difference between the SWS values reported by the two researchers reached 18.54% (EET, longitudinal plane) and was not statistically significant (*p* = 0.51) (Table 1). This observation advocates the reliability and repeatability of SWE in the diagnosis of neck structures. This high repeatability is what clearly promotes SWE over the SE technique, in which the interobserver agreement ranges from slight to fair [5,6]. Our results are not fully concordant with other reports, which provide inconsistent data. Lim et al. [22] provided results similar to ours and indicated significant interobserver and intraobserver agreement in SWE of thyroid nodules. Excellent interobserver reproducibility was also demonstrated by Veyrieres et al. [23], who evaluated thyroid nodules so as to find the SWE threshold for malignant lesions. On the other hand, in the paper by Swan et al. [24], the reproducibility of SWE of thyroid nodules was demonstrated as insufficient to reliably differentiate malignant nodules from benign nodules in an individual patient. Additionally, Kishimoto et al. [25] observed excellent interoperator reproducibility in phantoms, but it was insufficient for the thyroid and all other analyzed regions except for the liver in the healthy volunteers. Several important factors might generate this discrepancy. The most important factors are the influence of the surrounding tissues, including incorrect probe positioning (without an adequate gel thick coating, which should prevent pressure), anisotropy of the surrounding tissues, neck muscle tension or blood vessel pulsation [26,27].

In the present paper, significant differences between SWS assessed in the transverse and longitudinal planes were demonstrated in both ET and thyroid tissues. The same very high degree of statistical significance was achieved by both independent researchers (Table 1 and Table 2). However, all of the obtained quantitative results measured in both planes were within the values that are considered typical for benign lesions. Chen et al. [3] demonstrated a strong difference between SWS of benign and malignant thyroid lesions, with mean values of 19.2 ± 7.1 kPa and 34.6 ± 14.8 kPa, respectively. In that group, the mean SWS cut-off level equal to 24 kPa had a sensitivity of 78.8% and a specificity of 84.9% [3]. A similar cut-off level of 22.3 kPa was presented by Samir et al. [4], with sensitivity, specificity, positive predictive values and negative predictive values of 82%, 88%, 75% and 91%, respectively. The highest SWS values obtained in our study in the longitudinal plane for IETs, EETs and thyroid tissue were 16.5 kPa, 16.6 and 22.5 kPa, respectively (, and the maximum (among the two researchers) mean SWS values in the longitudinal plane were 13.99 ± 4.65, 11.15 ± 2.65 and 12.74 ± 2.80 kPa, respectively (Table 1 and Table 2). Thus, only one highest result reached the value close to the cut-off levels, suggested by the authors cited above [3,4], and the mean SWS values were much lower than any of the cut-offs. At the same time, the mean SWS values measured in the transverse plane did not reach 10 kPa for any of the analyzed structures (Table 1 and Table 2). The differences in SWS for malignant and benign lesions are particularly useful for the thyroid, as the most common thyroid malignancy is papillary thyroid carcinoma (PTC), which is distinctly stiffer than the normal thyroid tissue in SWE [11].

Despite the significant differences between SWS values in the transverse and longitudinal planes, the SWR values did not differ, regardless of the plane, and always indicated ETs as benign lesions whose stiffness was lower or equal to the thyroid stiffness (Table 3). These findings have proven that SWE is a useful method in the differential diagnosis of ETs, and the results are reliable despite the differences in SWS between the imaging planes. However, the question arises what the real reason for such differences is. To the best of our knowledge, no other publications analyzing this issue in regard to the neck structures are available, and thus we are not able to compare our results with those of other authors.

Quantitative elastography is based on the measurement of the shear wave propagation velocity and is sensitive to tissue anisotropy [27]. This phenomenon concerns tissues that have different microstructures in different planes. The shear wave speed is higher when the wave propagates parallel to the spatially oriented structures than perpendicularly to them. Therefore, when the ultrasound probe is placed parallel to these tissues, the shear wave propagates perpendicularly to them, and lower elasticity (higher SWS) values are observed. On the contrary, when the shear wave beam is emitted perpendicularly, it propagates at a higher speed, and higher elasticity values are generated. This property affects SWE values, as described before, particularly in studies concerning muscles, tendons and kidneys [27,28,29,30,31,32]. So far, this phenomenon has not been thoroughly described in the case of the thyroid gland, possibly because of the fact that the presence of anisotropy in thyroid tissue has not been confirmed on the basis of the apparent diffusion of the coefficient value in magnetic resonance examination [33]. Except for the present study, there is only one other published report analyzing SWS of the normal thyroid tissue in the two planes [34]. The authors of that paper mainly evaluated thyroid tissue altered in the course of Hashimoto’s thyroiditis (HT), and the normal thyroid measurements served as controls. They reported the differences in SWS between transverse and longitudinal sections, and these differences were even more pronounced for control tissues than HT, reaching about a 40% increase of SWS in the longitudinal plane [34]. These results are highly similar to ours. Unfortunately, the authors did not make any attempt to analyze their findings.

Importantly, in our study, these differences, presented as absolute (D) or percentage (D%) values, did not differ much between researchers (Table 2). This fact can suggest that the observed difference in SWS values between the planes is actually an artifact related to the fact that most of the organs of the neck are known to be anisotropic. Such a conclusion is supported by the results of Lee et al. [26], who provided SWS values of reactive cervical lymph nodes with respect to US probe position (parallel or perpendicular to the muscle fiber orientation) and with respect to the presence of muscle stretch stress. This study revealed that SWS values were higher in the parallel position and thereby confirmed the effect of the anisotropic nature of muscles on the SWS values of the cervical lymph nodes. This phenomenon was exacerbated by the stretch stress of the cervical muscles [26].

The awareness of such a phenomenon should probably result in the indication for a mandatory description, of which a cross-section type was applied for the particular SWS measurement in the neck tissues. The possible impact of this finding on diagnosing thyroid cancers remains to be elucidated. Prospective studies evaluating SWE values in thyroid focal lesions in both planes are therefore necessary.

Thus, on the basis of the results presented in the current paper, we postulate that the elasticity measurements can be influenced by the tissue architecture of the neck organs, and this phenomenon should be taken into account when analyzing SWS results for thyroid and other neck tissues. We believe that this phenomenon should not be considered an important limitation of the method, but rather an artifact related to the physical properties of the tissues. SWE was demonstrated to be an easy and quick diagnostic method, characterized by high intra- and interobserver agreement. This might easily result in uncritical acceptance of the obtained results, while our results have demonstrated that the cross-section should be taken into account when interpreting the SWS values. Understanding the possible influence of artifacts, resulting from different plane imaging, provides the basis for eliminating errors in the interpretation of SWE.

The study has a limitation concerning the study sample calculation. ETs are uncommon findings, so the study sample was collected by including all the consecutive patients with lesions meeting the FNAB criteria of ETs, without the sample size calculation.

In conclusion, SWE is characterized by high inter- and intraobserver agreement in the evaluation of ETs and adjacent thyroid tissue. However, the researchers should be aware of the presence of plane-dependent artifacts, resulting in higher SWS values in the longitudinal section. These artifacts do not influence the SWR values and do not reduce the reliability of the method in ETs diagnosis. In our opinion, the data gained from this research will improve the diagnosis, follow-up and management of the thyroid and neck lesions.

## Figures and Tables

**Table 1:** Comparison between SWS and SWR values obtained by two independent researchers.

	*n*	Researcher 1 Mean ± SD	Researcher 2 Mean ± SD	*p*	D Mean (95%CI)	D% Mean (95%CI)
**Transverse Plane**						
SWS of ET	50	7.23 ± 1.78	7.20 ± 1.54	0.84	−0.04 (−0.37; 0.30)	10.78 (7.24; 14.32)
SWS of EET	17	6.77 ± 1.39	7.09 ± 0.99	0.18	0.32 (−0.26; 0.90)	11.26 (5.32; 17.21)
SWS of IET	33	7.47 ± 1.93	7.25 ± 1.77	0.45	−0.22 (−0.63; 0.20)	10.53 (5.89; 15.17)
Thyroid SWS	33	8.66 ± 2.42	8.54 ± 2.23	0.97	−0.12 (−0.64; 0.39)	10.49 (6.30; 14.69)
SWR of IET	33	0.89 ± 0.21	0.88 ± 0.17	0.57	−0.01 (−0.06; 0.04)	11.3 0(8.01; 14.58)
**Longitudinal Plane**						
SWS of ET	49	11.64 ± 3.64	12.08 ± 3.64	0.10	0.43 (−0.12; 0.99)	11.53 (7.68; 15.38)
SWS of EET	16	13.45 ±4.30	13.99 ±4.65	0.51	0.54 (−1.09; 2.16)	18.54 (9.67; 27.40)
SWS of IET	33	10.77 ± 2.96	11.15 ± 2.65	0.051	0.38 (0.00; 0.77)	8.13 (4.53; 11.73)
Thyroid SWS	50	11.84 ± 3.24	12.09 ± 2.85	0.36	0.25 (−0.24; 0.74)	12.86 (9.24; 16.48)
SWS of thyroid adjacent to EET	17	11.08 ± 2.57	10.82 ± 2.60	0.59	−0.25 (−1.27; 0.77)	15.57 (8.29; 22.86)
SWS of thyroid adjacent to IET	33	12.23 ± 3.51	12.74 ± 2.80	0.13	0.52 (−0.03; 1.06)	11.46 (7.23; 15.69)
SWR of ET	49	0.86 ± 0.13	0.83 ± 0.12	0.12	−0.02 (−0.06; 0.01)	9.58 (6.32; 12.84)
SWR of EET	16	0.84 ± 0.15	0.81 ± 0.19	0.51	−0.03 (−0.12; 0.06)	13.75 (6.08; 21.41)
SWR of IET	33	0.87 ±0.12	0.85 ± 0.08	0.14	−0.02 (−0.06; 0.01)	7.57 (4.33; 10.80)

Abbreviations: CI, confidence interval; D, difference; EET, extrathyroidal ectopic thymuses; ET, ectopic thymuses; IET, intrathyroidal ectopic thymuses; SD, standard deviation; SWR, shear wave ratio; SWS, shear wave stiffness.

**Table 2:** Comparison of SWS values of ectopic thymuses measured in the transverse and longitudinal planes.

Ectopic Thymus	*n*	Transverse SWS Mean ± SD	Longitudinal SWS Mean ± SD	*p*	D Mean (95%CI)	D% Mean (95%CI)
Researcher 1						
ETs	49	7.23 ± 1.78	11.64 ± 3.64	*<0.0000001*	4.40 (3.29; 5.52)	46.47 (38.08; 54.87)
EETs	16	6.77 ± 1.39	13.45 ±4.30	*<0.001*	6.68 (4.46; 8.90)	62.21 (48.29; 76.13)
IETs	33	7.47 ± 1.93	10.77 ± 2.96	*<0.0001*	3.30 (2.14; 4.45)	38.84 (28.95; 48.73)
Researcher 2						
ETs	49	7.20 ± 1.54	12.08 ± 3.64	*<0.00000001*	4.88 (3.85; 5.90)	47.62 (39.87; 55.37)
EETs	16	7.09 ± 0.99	13.99 ± 4.65	*<0.001*	6.89 (4.60; 9.17)	60.65 (47.02; 74.27)
IETs	33	7.25 ± 1.77	11.15 ± 2.65	*<0.000001*	3.90 (2.94; 4.86)	41.31 (32.21; 50.40)

Abbreviations: CI, confidence interval; D, difference; EETs, extrathyroidal ectopic thymuses; IET, intrathyroidal ectopic thymuses; SD, standard deviation; SWS, shear wave stiffness.

**Table 3:** Comparison of the thyroid SWS values measured in the transverse and longitudinal planes.

Thyroid	*n*	Transverse SWS Mean ± SD	Longitudinal SWS Mean ± SD	*p*	D Mean (95%CI)	D% Mean (95%CI)
**R1**	33	8.66 ± 2.42	12.23 ± 3.51	*0.0001*	3.57 (2.15; 4.98)	39.69 (29.86; 49.53)
**R2**	33	8.54 ± 2.23	12.74 ± 2.80	*<0.00001*	4.21 (3.05; 5.36)	40.79 (31.53; 50.06)

Abbreviations: CI, confidence interval; D, difference; SD, standard deviation; SWS, shear wave stiffness; R1, researcher 1; R2, researcher 2.

**Table 4:** Comparison of the SWR values measured in the transverse and longitudinal planes.

	*n*	Transverse SWR Mean ± SD	Longitudinal SWR Mean ± SD	*p*	D Mean (95%CI)	D% Mean (95%CI)
**R1**	33	0.89 ± 0.21	0.87 ± 0.12	*0.79*	−0.02 (−0.11; 0.07)	22.01 (15.57; 28.46)
**R2**	33	0.88 ± 0.17	0.85 ± 0.08	*0.41*	−0.03 (−0.09; 0.02)	13.99 (9.58; 18.41)

Abbreviations: CI, confidence interval; D, difference; SD, standard deviation; SWR, shear wave ratio; R1, researcher 1; R2, researcher 2.

## Abbreviations

CI	confidence interval
D	difference
EET	extrathyroidal ectopic thymic tissue;
ET	ectopic thymus;
FNAB	fine-needle aspiration biopsy
IET	intrathyroidal ectopic thymus;
PD	power Doppler;
PTC	papillary thyroid carcinoma;
R1	researcher 1
R2	researcher 2
SD	standard deviation
SE	strain elastography
SWE	shear wave elastography
SWS	shear wave stiffness
SWR	shear wave ratio
US	ultrasound
